# Supplementary material for: Detailed molecular and epigenetic characterization of the pig IPEC-J2 and chicken SL-29 cell lines
Source: iScience. 2023 Feb 20;26(3):106252. doi: 10.1016/j.isci.2023.106252 (PMC10018572; doi:10.1016/j.isci.2023.106252)
Supplement: Data S2. Complete homer output for identified motifs in Chicken SL-29, related to Tables 5 and 6 — Homer motif analysis results for histone modifications H3K4me1, H3K4me3, H3K27ac, enhancers, and ATAC-seq of chicken SL-29 cell line. Parameters for possible false positives is as mentioned earlier for S5. [file mmc3.zip › Data_S2/S6/Chicken_SL_29/peak_files_H3K4me1/homerResults.html]

peak\_files\_H3K4me1/ - Homer de novo Motif Results


# Homer *de novo* Motif Results (peak\_files\_H3K4me1/)

Known Motif Enrichment Results  
Gene Ontology Enrichment Results  
If Homer is having trouble matching a motif to a known motif, try copy/pasting the matrix file into
STAMP  
More information on motif finding results: HOMER
| Description of Results
| Tips
  
Total target sequences = 49873  
Total background sequences = 49955  
\* - possible false positive  

|  |  |  |  |  |  |  |  |  |
| --- | --- | --- | --- | --- | --- | --- | --- | --- |
| Rank | Motif | P-value | log P-pvalue | % of Targets | % of Background | STD(Bg STD) | Best Match/Details | Motif File |
| 1 | T C G A A G C T C T A G C G T A T A C G A C G T G T A C T C G A A G C T G T C A | 1e-89 | -2.064e+02 | 3.93% | 2.42% | 97.9bp (86.3bp) | Atf3(bZIP)/GBM-ATF3-ChIP-Seq(GSE33912)/Homer(0.973) More Information \| Similar Motifs Found | motif file (matrix) |
| 2 | A G C T A G T C A T C G C G A T A C T G A G T C A G T C G C A T A C T G A C T G G T A C G A C T A G T C | 1e-42 | -9.680e+01 | 0.09% | 0.01% | 85.1bp (55.4bp) | Pax8(Paired,Homeobox)/Thyroid-Pax8-ChIP-Seq(GSE26938)/Homer(0.646) More Information \| Similar Motifs Found | motif file (matrix) |
| 3 | G C T A G C A T G C T A A G C T C G T A C T G A A G T C C G A T C A G T C A T G C A T G C T A G T C A G | 1e-42 | -9.671e+01 | 0.07% | 0.00% | 80.0bp (20.4bp) | PSE(SNAPc)/K562-mStart-Seq/Homer(0.565) More Information \| Similar Motifs Found | motif file (matrix) |
| 4 | A C T G C G T A A C T G A G T C A C G T A T C G C G A T A C T G G T C A A G T C G T C A A G T C C T A G | 1e-38 | -8.957e+01 | 0.07% | 0.00% | 94.1bp (9.3bp) | TBX15/MA0803.1/Jaspar(0.653) More Information \| Similar Motifs Found | motif file (matrix) |
| 5 | G A T C G A C T A G T C C G T A A C T G A G T C G A T C G A T C G T A C A C G T A T G C C T A G A G C T | 1e-35 | -8.254e+01 | 0.06% | 0.00% | 88.9bp (0.0bp) | ZNF682/MA1599.1/Jaspar(0.627) More Information \| Similar Motifs Found | motif file (matrix) |
| 6 | C G T A A C G T C G A T A C T G C G A T T A C G G T C A C A G T T C G A C A T G A T C G C G A T C G T A | 1e-35 | -8.254e+01 | 0.06% | 0.00% | 82.1bp (0.0bp) | PB0168.1\_Sox14\_2/Jaspar(0.604) More Information \| Similar Motifs Found | motif file (matrix) |
| 7 | A T G C A T C G A G C T C A G T C A G T C T A G C A T G T A C G C G T A C T A G | 1e-33 | -7.688e+01 | 40.33% | 37.69% | 84.1bp (86.4bp) | WT1(Zf)/Kidney-WT1-ChIP-Seq(GSE90016)/Homer(0.665) More Information \| Similar Motifs Found | motif file (matrix) |
| 8 | A T C G A C T G C A T G C T A G T G A C G C A T A C T G A G T C A G T C G A T C | 1e-32 | -7.576e+01 | 11.18% | 9.56% | 83.5bp (87.6bp) | NFkB-p50,p52(RHD)/Monocyte-p50-ChIP-Chip(Schreiber\_et\_al.)/Homer(0.753) More Information \| Similar Motifs Found | motif file (matrix) |
| 9 | C T A G C T A G A T C G G T A C G T C A C T G A C G T A A T G C G A C T A C G T A G C T G T C A T A G C | 1e-32 | -7.565e+01 | 0.07% | 0.00% | 85.5bp (61.4bp) | PB0141.1\_Isgf3g\_2/Jaspar(0.648) More Information \| Similar Motifs Found | motif file (matrix) |
| 10 | A G T C C G A T G T C A C T G A A C T G A C G T A C T G A C G T A C T G C A T G A T C G A C G T A C G T | 1e-31 | -7.224e+01 | 0.06% | 0.00% | 76.7bp (0.0bp) | FOXH1/MA0479.1/Jaspar(0.660) More Information \| Similar Motifs Found | motif file (matrix) |
| 11 | C G A T T A C G A C T G C T A G G A C T C G A T G C A T A C G T C A G T G T C A | 1e-30 | -7.019e+01 | 29.56% | 27.23% | 84.7bp (86.1bp) | PH0078.1\_Hoxd13/Jaspar(0.600) More Information \| Similar Motifs Found | motif file (matrix) |
| 12 | A C T G G T C A C T G A A T C G C G T A A C T G A G T C A G T C A G T C A G T C A C T G A C G T C A G T | 1e-29 | -6.887e+01 | 0.06% | 0.00% | 87.5bp (23.6bp) | ZNF341(Zf)/EBV-ZNF341-ChIP-Seq(GSE113194)/Homer(0.660) More Information \| Similar Motifs Found | motif file (matrix) |
| 13 | C G A T A G T C G A T C G C A T A G C T C G T A T C A G C G T A C A T G C G A T G A T C G C T A G A T C | 1e-29 | -6.697e+01 | 0.07% | 0.01% | 78.8bp (47.3bp) | BATF3/MA0835.2/Jaspar(0.573) More Information \| Similar Motifs Found | motif file (matrix) |
| 14 | G T C A C T G A T A G C T A C G G C T A C A G T C T A G A T C G C G T A T C A G C A G T C T A G C T G A | 1e-29 | -6.697e+01 | 0.07% | 0.01% | 84.6bp (32.1bp) | Srebp1a(bHLH)/HepG2-Srebp1a-ChIP-Seq(GSE31477)/Homer(0.743) More Information \| Similar Motifs Found | motif file (matrix) |
| 15 | G A C T G T C A G A C T C T G A T G A C G C T A T G A C C G T A C A T G C T A G T C A G T G C A T G A C | 1e-29 | -6.697e+01 | 0.07% | 0.01% | 78.8bp (99.6bp) | POL009.1\_DCE\_S\_II/Jaspar(0.657) More Information \| Similar Motifs Found | motif file (matrix) |
| 16 | T G A C G C T A A T C G G T C A A C T G A G T C A G C T A C T G T A C G A G C T C A T G G A T C A T G C | 1e-28 | -6.673e+01 | 0.08% | 0.01% | 83.7bp (58.6bp) | Tcf12(bHLH)/GM12878-Tcf12-ChIP-Seq(GSE32465)/Homer(0.670) More Information \| Similar Motifs Found | motif file (matrix) |
| 17 | G C T A T G C A A T G C C G A T T G A C T C G A A C T G T G C A T A C G T A G C | 1e-26 | -6.209e+01 | 39.77% | 37.41% | 84.6bp (87.7bp) | Gfi1b/MA0483.1/Jaspar(0.655) More Information \| Similar Motifs Found | motif file (matrix) |
| 18 | C A T G G A C T G C T A C G A T A C G T T C G A G A T C G C A T A T G C C G T A C A T G A G C T A G T C | 1e-26 | -6.133e+01 | 0.06% | 0.01% | 94.8bp (57.8bp) | BATF3/MA0835.2/Jaspar(0.693) More Information \| Similar Motifs Found | motif file (matrix) |
| 19 | G T C A T A C G A G T C C G T A A C T G A C T G C G T A C G T A | 1e-26 | -6.016e+01 | 17.49% | 15.72% | 86.2bp (87.6bp) | IKZF1/MA1508.1/Jaspar(0.863) More Information \| Similar Motifs Found | motif file (matrix) |
| 20 | C A T G C G T A A C T G C T G A A C T G T A G C C G A T A C T G C G T A A C G T G T A C C A T G A C G T | 1e-25 | -5.898e+01 | 0.05% | 0.00% | 78.5bp (0.0bp) | ATOH1(var.2)/MA1467.1/Jaspar(0.622) More Information \| Similar Motifs Found | motif file (matrix) |
| 21 | G A T C C T A G T G A C G C T A A C T G T G A C G T A C G C T A A T G C G A T C G A C T A T G C G A C T | 1e-25 | -5.799e+01 | 0.11% | 0.02% | 78.2bp (68.0bp) | ZNF7(Zf)/HepG2-ZNF7.Flag-ChIP-Seq(Encode)/Homer(0.603) More Information \| Similar Motifs Found | motif file (matrix) |
| 22 | A T G C C T G A G T C A C G T A A C G T T C G A C A T G C A T G | 1e-24 | -5.538e+01 | 16.06% | 14.43% | 84.4bp (86.2bp) | MXI1/MA1108.2/Jaspar(0.674) More Information \| Similar Motifs Found | motif file (matrix) |
| 23 | G A T C A G T C G A T C A G T C A T C G C G A T T A C G C T A G C A T G C T A G | 1e-22 | -5.280e+01 | 14.11% | 12.61% | 83.0bp (84.5bp) | PB0102.1\_Zic2\_1/Jaspar(0.763) More Information \| Similar Motifs Found | motif file (matrix) |
| 24 | G C T A C T G A G A T C G A C T A G C T G C T A T G A C G C T A C T A G A T C G G T A C G C A T C T G A | 1e-22 | -5.257e+01 | 0.05% | 0.00% | 87.0bp (0.0bp) | PBX1(Homeobox)/MCF7-PBX1-ChIP-Seq(GSE28007)/Homer(0.559) More Information \| Similar Motifs Found | motif file (matrix) |
| 25 | G C T A A T G C A C G T C G A T A C T G A G C T A G T C A G C T C G T A A T C G A C G T A C T G A T G C | 1e-22 | -5.257e+01 | 0.05% | 0.00% | 85.5bp (0.0bp) | Smad4/MA1153.1/Jaspar(0.632) More Information \| Similar Motifs Found | motif file (matrix) |
| 26 | C G T A A G T C C G T A A G T C A C T G C G T A A G T C A C G T C G A T A T G C | 1e-22 | -5.172e+01 | 0.07% | 0.01% | 91.0bp (72.7bp) | Npas4(bHLH)/Neuron-Npas4-ChIP-Seq(GSE127793)/Homer(0.734) More Information \| Similar Motifs Found | motif file (matrix) |
| 27 | C G T A T G C A T A G C G A C T G T A C G T A C T C A G T G A C G C T A A T C G G C T A A C T G T A G C | 1e-22 | -5.172e+01 | 0.07% | 0.01% | 83.1bp (47.4bp) | POL013.1\_MED-1/Jaspar(0.524) More Information \| Similar Motifs Found | motif file (matrix) |
| 28 | C G T A T A C G G A T C C T A G C A G T A C T G T G A C C G A T A T C G A G T C A C T G A T G C C G A T | 1e-20 | -4.778e+01 | 0.05% | 0.01% | 77.5bp (86.4bp) | PB0207.1\_Zic3\_2/Jaspar(0.688) More Information \| Similar Motifs Found | motif file (matrix) |
| 29 | C T A G G C A T A C T G A C G T T C G A G A C T G A C T A C G T | 1e-19 | -4.513e+01 | 16.39% | 14.91% | 85.9bp (87.5bp) | Foxh1(Forkhead)/hESC-FOXH1-ChIP-Seq(GSE29422)/Homer(0.724) More Information \| Similar Motifs Found | motif file (matrix) |
| 30 | A G T C T A G C G A T C T A G C G C T A A C T G A G T C T G C A | 1e-19 | -4.470e+01 | 23.74% | 22.03% | 83.6bp (88.9bp) | Zic1::Zic2/MA1628.1/Jaspar(0.735) More Information \| Similar Motifs Found | motif file (matrix) |
| 31 | T C A G T G C A T C A G A G T C A C T G G A T C A C G T C A T G | 1e-18 | -4.235e+01 | 35.70% | 33.81% | 85.3bp (89.6bp) | POL002.1\_INR/Jaspar(0.617) More Information \| Similar Motifs Found | motif file (matrix) |
| 32 | A G C T C T G A G T C A G A T C T C A G G A T C A G C T A T C G | 1e-17 | -4.019e+01 | 11.30% | 10.12% | 85.1bp (89.0bp) | OVOL1/MA1544.1/Jaspar(0.652) More Information \| Similar Motifs Found | motif file (matrix) |
| 33 | A C G T C G T A A G T C C G T A A C G T A C T G A C T G A C T G A G T C A G T C A C G T A G T C C G T A | 1e-17 | -4.008e+01 | 0.05% | 0.00% | 79.1bp (52.2bp) | Zac1(Zf)/Neuro2A-Plagl1-ChIP-Seq(GSE75942)/Homer(0.634) More Information \| Similar Motifs Found | motif file (matrix) |
| 34 | T A C G C T A G A C T G G T C A C G T A A C G T A C T G A G T C | 1e-16 | -3.889e+01 | 11.96% | 10.76% | 85.2bp (86.9bp) | TEAD4(TEA)/Tropoblast-Tead4-ChIP-Seq(GSE37350)/Homer(0.846) More Information \| Similar Motifs Found | motif file (matrix) |
| 35 | C G T A C G T A A C G T C G T A A G T C A C T G A C G T C G T A A C G T A C G T | 1e-16 | -3.759e+01 | 0.05% | 0.01% | 91.7bp (57.7bp) | PB0106.1\_Arid5a\_2/Jaspar(0.713) More Information \| Similar Motifs Found | motif file (matrix) |
| 36 | A G T C A C T G A G C T A C G T A G T C G T A C A G T C C G T A | 1e-15 | -3.606e+01 | 3.18% | 2.58% | 83.0bp (90.3bp) | RBPJ/MA1116.1/Jaspar(0.744) More Information \| Similar Motifs Found | motif file (matrix) |
| 37 | A C T G C G T A A G T C A C G T C G T A A T C G A C T G A G T C A C G T A C G T | 1e-15 | -3.559e+01 | 0.05% | 0.01% | 92.4bp (41.6bp) | ZFX(Zf)/mES-Zfx-ChIP-Seq(GSE11431)/Homer(0.643) More Information \| Similar Motifs Found | motif file (matrix) |
| 38 | C G T A C G T A A G T C A G T C A C T G A G C T A C T G C G A T A G T C A C G T | 1e-14 | -3.294e+01 | 0.06% | 0.01% | 88.5bp (63.6bp) | MF0009.1\_TRP(MYB)\_class/Jaspar(0.634) More Information \| Similar Motifs Found | motif file (matrix) |
| 39 | C G A T A C G T G A T C A G C T C A G T G T C A C T A G A C T G | 1e-14 | -3.228e+01 | 16.96% | 15.70% | 85.2bp (86.8bp) | STAT6(Stat)/Macrophage-Stat6-ChIP-Seq(GSE38377)/Homer(0.743) More Information \| Similar Motifs Found | motif file (matrix) |
| 40 | A C G T C G A T A C G T A G T C C G T A C G T A A G T C A C T G A C G T A G T C | 1e-13 | -3.038e+01 | 0.04% | 0.01% | 76.6bp (40.3bp) | ESRRB/MA0141.3/Jaspar(0.675) More Information \| Similar Motifs Found | motif file (matrix) |
| 41 | A G T C C G T A A G T C A C T G A C G T C G T A C G T A A C T G A C T G C G T A | 1e-12 | -2.807e+01 | 0.04% | 0.01% | 84.8bp (16.1bp) | HIF-1a(bHLH)/MCF7-HIF1a-ChIP-Seq(GSE28352)/Homer(0.712) More Information \| Similar Motifs Found | motif file (matrix) |
| 42 \* | A G T C A G T C A C T G A C G T A C T G A G T C C G T A C G T A A C G T A C T G | 1e-11 | -2.719e+01 | 0.05% | 0.01% | 74.6bp (81.7bp) | PB0199.1\_Zfp161\_2/Jaspar(0.690) More Information \| Similar Motifs Found | motif file (matrix) |
| 43 \* | A C G T A C T G C G T A A C G T A C G T C G A T C G T A C G T A | 1e-11 | -2.606e+01 | 5.74% | 5.06% | 87.3bp (88.4bp) | Chop(bZIP)/MEF-Chop-ChIP-Seq(GSE35681)/Homer(0.797) More Information \| Similar Motifs Found | motif file (matrix) |
| 44 \* | A G T C A C T G C G T A A C T G C G T A A G T C C G T A A C T G A C T G C G T A | 1e-11 | -2.581e+01 | 0.04% | 0.01% | 60.1bp (82.7bp) | PB0060.1\_Smad3\_1/Jaspar(0.697) More Information \| Similar Motifs Found | motif file (matrix) |
| 45 \* | A C T G C G T A C G T A A C T G G T C A C G T A C G T A A C G T A G T C A C T G | 1e-11 | -2.581e+01 | 0.04% | 0.00% | 70.4bp (41.6bp) | PH0037.1\_Hdx/Jaspar(0.663) More Information \| Similar Motifs Found | motif file (matrix) |
| 46 \* | A T C G G A C T C A G T G T C A G A C T A C G T G C A T A G T C | 1e-9 | -2.198e+01 | 8.18% | 7.44% | 84.6bp (86.8bp) | PB0119.1\_Foxa2\_2/Jaspar(0.694) More Information \| Similar Motifs Found | motif file (matrix) |
| 47 \* | A C G T C T A G A C T G C G T A A C G T A G T C A C G T A C G T | 1e-9 | -2.105e+01 | 1.99% | 1.63% | 85.1bp (86.5bp) | TCF7L1/MA1421.1/Jaspar(0.696) More Information \| Similar Motifs Found | motif file (matrix) |
| 48 \* | A G T C A G T C A C T G A C G T A G T C A C T G A C G T C G T A | 1e-8 | -1.937e+01 | 0.03% | 0.01% | 76.1bp (95.0bp) | PH0077.1\_Hoxd12/Jaspar(0.691) More Information \| Similar Motifs Found | motif file (matrix) |
| 49 \* | A G T C C G T A A G T C A C T G A G T C A C G T C G T A C G T A | 1e-7 | -1.638e+01 | 0.55% | 0.39% | 81.0bp (91.4bp) | Ahr::Arnt/MA0006.1/Jaspar(0.777) More Information \| Similar Motifs Found | motif file (matrix) |
| 50 \* | A G T C A G T C A C G T A C T G A G T C A C T G A C T G A C G T A C T G A C T G | 1e-5 | -1.361e+01 | 0.06% | 0.03% | 68.5bp (109.2bp) | Zic2/MA1629.1/Jaspar(0.757) More Information \| Similar Motifs Found | motif file (matrix) |
| 51 \* | C T G A A G T C A C G T A C G T A C T G A C T G A C T G A G C T A C T G A C T G | 1e-4 | -1.123e+01 | 0.14% | 0.08% | 90.9bp (41.9bp) | GLI3(Zf)/Limb-GLI3-ChIP-Chip(GSE11077)/Homer(0.741) More Information \| Similar Motifs Found | motif file (matrix) |
| 52 \* | C G T A A G T C C G T A A G T C A G T C A C T G A G T C A C T G | 1e0 | -1.499e+00 | 0.12% | 0.11% | 67.8bp (0.0bp) | TBX3/MA1566.1/Jaspar(0.698) More Information \| Similar Motifs Found | motif file (matrix) |
